# Supplementary material for: Dynalign II: common secondary structure prediction for RNA homologs with domain insertions
Source: Nucleic Acids Res. 2014 Nov 21;42(22):13939–48. doi: 10.1093/nar/gku1172 (PMC4267632; doi:10.1093/nar/gku1172)
Supplement: SUPPLEMENTARY DATA [file supp_gku1172_nar-02021-z-2014-File012.zip › manual/RNA_class/html/functions_vars.html]

RNAstructure Classes: Class Members - Variables

- Main Page
- Classes
- Files

- Class List
- Class Hierarchy
- Class Members

- All
- Functions
- Variables
- Typedefs

- a
- c
- d
- e
- f
- g
- h
- i
- l
- m
- n
- o
- p
- q
- r
- s
- t
- v
- w

### - a -

- aliFiles
  : Multilign\_object- align
    : Dynalign\_object- array
      : Dynalign\_object

### - c -

- compoundmessage
  : TwoRNA- ct
    : RNA- ct\_files
      : Multilign\_object- ct\_templated
        : Dynalign\_object

### - d -

- data
  : Dynalign\_object
  , Thermodynamics- dGIndex
    : Multilign\_object- drawallocated
      : RNA- dsv\_templated
        : Dynalign\_object- dsvFiles
          : Multilign\_object

### - e -

- energies
  : Multilign\_object- energyallocated
    : RNA- energyread
      : Thermodynamics- enthalpy
        : Thermodynamics- ErrorCode
          : Multilign\_object
          , RNA- ErrorCodeTwo
            : TwoRNA- ev
              : RNA- ew
                : RNA- ew2
                  : RNA- ew3
                    : RNA- ew5
                      : RNA- ewmb
                        : RNA- ewmb2
                          : RNA

### - f -

- fce
  : RNA- forbidunimolecular
    : HybridRNA- forcealign
      : Dynalign\_object

### - g -

- gap
  : Dynalign\_object

### - h -

- highend
  : Dynalign\_object

### - i -

- input\_alignment
  : Multilign\_object- input\_sequences
    : Multilign\_object- inputList
      : Multilign\_object- instance
        : Multilign\_object- isrna
          : Thermodynamics- isRNA
            : Multilign\_object- iterations
              : Multilign\_object

### - l -

- length
  : Oligowalk\_object- lfce
    : RNA- lowend
      : Dynalign\_object- lowest
        : Dynalign\_object

### - m -

- MAXDSV
  : Dynalign\_object- maxDsv
    : Multilign\_object- maxPairs
      : Multilign\_object- Maxtrace
        : Dynalign\_object- mod
          : RNA- modificationflag
            : Dynalign\_object

### - n -

- num\_processors
  : Multifind\_object- numofsubstructures
    : Oligowalk\_object

### - o -

- output\_multifind
  : Multilign\_object

### - p -

- pair\_alignments
  : Multilign\_object- partitionfunctionallocated
    : RNA- pfdata
      : RNA- prefilter
        : Oligowalk\_object- progress
          : Multilign\_object
          , RNA

### - q -

- Q
  : RNA

### - r -

- rna1
  : TwoRNA- rna2
    : TwoRNA

### - s -

- savefileread
  : Dynalign\_object- seqPair
    : Multilign\_object- sequences
      : HybridRNA- SHAPEIntercept
        : Multilign\_object- SHAPESlope
          : Multilign\_object- structurecoordinates
            : RNA

### - t -

- table
  : Oligowalk\_object- temp
    : Thermodynamics- temperature
      : Multilign\_object- templatefilename
        : Dynalign\_object

### - v -

- v
  : Dynalign\_object
  , RNA- vmin
    : RNA- vmod
      : Dynalign\_object

### - w -

- w
  : Dynalign\_object
  , RNA- w3
    : RNA
    , Dynalign\_object- w5
      : Dynalign\_object
      , RNA- wca
        : RNA- wcoax
          : RNA- wl
            : RNA- wmb
              : RNA- wmbl
                : RNA

---

Generated on Tue Jul 30 10:52:29 2013 for RNAstructure Classes by 
 1.5.7.1
